# Why the randomized controlled trial is still at the apex and the gold standard for evaluating new medical and surgical interventions

**DOI:** 10.2340/17453674.2025.44947

**Published:** 2025-11-25

**Authors:** Aleksi Reito, Søren Overgaard

**Affiliations:** Center for Musculoskeletal Diseases, Tampere University Hospital, Tampere, Coxa Hospital for Joint Replacement, Tampere, Finland Copenhagen University Hospital Bispebjerg; Department of Orthopaedic Surgery and Traumatology and Department of Clinical Medicine, University of Copenhagen, Denmark

Randomized controlled trials (RCTs) sit at the apex of the evidence hierarchy because random allocation minimizes systemic confounding caused by both known and unknown confounders, allowing investigators to estimate the causal effect of an intervention. Their importance is universally acknowledged in the pharmaceutical industry in drug development, yet the same methodological rigor is occasionally overlooked when evaluating surgical procedures and implantable devices in orthopedic surgery and traumatology. That inconsistency is increasingly untenable.

Surgical techniques and implants often diffuse into clinical practice based on limited evidence, such as observational case series or even technical notes, driven by the desire to participate in innovation. In doing so, both surgeon and patient effectively become part of an uncontrolled experiment. In orthopedic surgery and traumatology, we have had tangible experience of how widely adopted orthopedic implants were later withdrawn after RCTs or registry data revealed inferior performance [1].

History has shown that the effects of incremental design changes cannot be exactly predicted from simulator or biomechanical studies, and changes, although marginal, may alter safety profiles in ways that even seasoned surgeons cannot anticipate. Experimental studies, though important for testing, cannot mimic the unprecedented behavior in the human body. Moreover, often the manufacturers promise better clinical outcomes, like a better range of motion and patient-reported outcomes, enhanced recovery, and even reduced complication risk. Often, these statements are not based on any evidence other than the experts involved in the development of an implant or new technique. This is obviously not sufficient for starting to use a new implant or technology like e.g., robotic surgery. Only randomized comparisons can disentangle the efficacy of the device or technique itself from variations in surgical skill, rehabilitation protocols, patient selection, and other factors that may bias an enthusiastic surgeon.

Ethics also favor randomization. When genuine uncertainty (clinical equipoise) exists about which treatment is superior, withholding randomization exposes half the patients to a potentially inferior intervention without the prospect of generating definitive knowledge. Even if hip simulator studies show excellent performance and biomechanical reasoning gives a robust basis for new implant design, they do not resolve the problem of how it would behave in the clinical setting. In contrast, an RCT ensures that every participant contributes to a collective answer that will guide future patients and the surgeon. Regulatory agencies have begun to echo this stance: both the U.S. FDA’s Breakthrough Devices Program and the EU Medical Device Regulation emphasize the need for high-quality comparative evidence before widespread adoption [2].

While observational studies and registry analyses remain valuable for hypothesis generation and post-market surveillance, they cannot replace evidence generated from randomized trials. Techniques such as propensity scoring, instrumental variables, and target-trial emulation improve causal inference but still rest on unverifiable assumptions about unmeasured confounding [3,4]. Importantly, although a single well-conducted RCT can provide compelling evidence, the strongest conclusions come from multiple independent trials that allow meta-analysis, exploration of heterogeneity, and confirmation that results are not driven by chance or context-specific biases. One RCT is good; a coherent body of several RCTs is the gold standard given that the trials have been well performed.

In the light of all the above, *Acta Orthopaedica* was pleased to publish 2 RCTs investigating newly introduced total knee replacement implant systems [5,6]. Newer implant systems are increasingly claimed to allow personalization of the implant choice. This description carries an implicit claim of improvement and better outcomes as personalization is generally very popular term in modern medicine and always associated with the proposition of improving patient care. These 2 studies were rigorous RCTs comparing the new Persona knee system with earlier implant design with proven track-record. Most importantly, these studies were independent studies done by independent researchers not involved in designing the implants or the technique. These trials provided consistent evidence that the Persona knee system does not provide any patient-relevant benefit in terms of functional outcome in short-term follow-up compared with the older implant with a proven track record and well-established clinical outcomes. Both studies also involved the same outcome assessment. This is a textbook example of how new innovations should be tested before wider adoption, but these implants are in fact already in routine clinical use.

RCTs can also serve as a platform for spin-off studies generating evidence for topics which are rarely primary outcomes in randomized studies. An excellent example are randomized trials investigating primarily total knee implants’ performance, but the secondary aim includes assessment of radiostereometric measurements (RSA) [7,8]. While a clinical trial involving only RSA measurement may not be considered worthwhile to conduct, it serves as an excellent spin-off study carried alongside more patient-focused clinical trials evaluating the risk of revision.

Although RCT’s are said to be the ultimate study design, there is no inherent guarantee that an RCT is of good quality. Initiating this kind of trial requires a team of researchers with the right qualifications taking also into account who is the sponsor of the study. This is from the very first step of getting an idea and defining the research question, to reporting the trial, which includes fulfilling of several requirements [9].

Acta’s editorial team warmly encourages you to submit your clinical trials. Our dedicated team of experienced reviewers is committed to providing a thorough, constructive, and high-quality assessment of your study.

Complete authors disclosure of interest forms according to ICMJE are available on the article page, doi: 10.2340/17453674.2025.44947


**Aleksi Reito** *Center for Musculoskeletal Diseases, Tampere University Hospital, Tampere* *Coxa Hospital for Joint Replacement, Tampere, Finland* *E-mail: aleksi@reito.fi*
**Søren Overgaard** *Copenhagen University Hospital Bispebjerg* *Department of Orthopaedic Surgery and Traumatology* *and* *Department of Clinical Medicine, University of Copenhagen, Denmark*

